# ASO Author Reflections: Young Women with Breast Cancer may Safely Opt for Breast Conservation After Neoadjuvant Therapy

**DOI:** 10.1245/s10434-026-20250-x

**Published:** 2026-07-17

**Authors:** J. Tseng, J. C. Boughey, R. A. Mukhtar

**Affiliations:** 1 City of Hope Comprehensive Cancer Center, Irvine, CA USA; 2 Mayo Clinic, Rochester, MN USA; 3 University of California San Francisco, San Francisco, CA USA

## Past

Young women with breast cancer undergo mastectomy at higher rates than older women, even when breast-conserving surgery (BCS) is feasible. Concerns regarding recurrence, hereditary cancer syndromes, and long-term outcomes often influence surgical decision-making. Although prior studies have demonstrated equivalent survival with BCS and mastectomy for appropriately selected patients with early-stage breast cancer, younger women have been underrepresented, and data in the modern neoadjuvant systemic therapy (NST) era remain limited.^1^^,^^2^

Neoadjuvant systemic therapy has expanded eligibility for breast conservation by downstaging disease; yet uncertainty persists regarding the impact of age on surgical decision-making after treatment. We therefore evaluated patterns of breast surgery and associated oncologic outcomes among young women enrolled in the I-SPY2 neoadjuvant clinical trial, a multicenter study of patients with high-risk stage II–III breast cancer.

## Present

Among 1737 patients enrolled in I-SPY2, women aged ≤45 years were significantly less likely to undergo BCS than older women despite similar tumor characteristics and responses to NST.^3^ However, rates of BCS in younger women increased steadily from 2010 to 2022, reflecting evolving surgical practice patterns.

Most importantly, the extent of breast surgery was not associated with locoregional recurrence-free interval or overall survival among younger women, even in the subgroup aged ≤40 years. In contrast, Residual Cancer Burden (RCB) after NST was strongly associated with recurrence and survival outcomes. These findings suggest that tumor biology and treatment response are more important determinants of outcome than the choice of mastectomy versus BCS. For appropriately selected young women, age alone should not be considered an indication for mastectomy.

## Future

Future research should focus on understanding why mastectomy rates remain high among young women despite evidence supporting the oncologic safety of breast conservation. Greater insight into patient preferences, perceived recurrence risk, genetic factors, surveillance strategies, and physician recommendations will help guide shared decision-making.

Additional studies incorporating germline testing results, contralateral prophylactic mastectomy rates, and long-term survivorship outcomes are needed. As these data mature within I-SPY2, they may better define factors influencing surgical choices and outcomes. Ultimately, these findings provide reassurance that tumor biology and response to treatment should guide treatment recommendations for young women rather than age alone.
